# Human antimicrobial protein hCAP18/LL-37 promotes a metastatic phenotype in breast cancer

**DOI:** 10.1186/bcr2221

**Published:** 2009-01-30

**Authors:** Günther Weber, Clara Ibel Chamorro, Fredrik Granath, Annelie Liljegren, Sami Zreika, Zuzana Saidak, Bengt Sandstedt, Samuel Rotstein, Romuald Mentaverri, Fabio Sánchez, Andor Pivarcsi, Mona Ståhle

**Affiliations:** 1 GICC, UMR CNRS 6239, Université François Rabelais, Avenue Monge, Tours, 37 100, France; 2 Unit of Dermatology and Venereology, Department of Medicine, Karolinska Institutet, CMM:02, Stockholm, 171 76, Sweden; 3 Department of Oncology and Pathology, Karolinska Institutet, Stockholm, 171 76, Sweden; 4 INSERM, ERI-12, Faculty of Pharmacy, 1 Rue des Louvels, Amiens, 80 037, France

## Abstract

**Introduction:**

Human cathelicidin antimicrobial protein, hCAP18, and its C-terminal peptide LL-37 is a multifunctional protein. In addition to being important in antimicrobial defense, it induces chemotaxis, stimulates angiogenesis and promotes tissue repair. We previously showed that human breast cancer cells express high amounts of hCAP18, and hypothesised that hCAP18/LL-37 may be involved in tumour progression.

**Methods:**

*hCAP18 *mRNA was quantified in 109 primary breast cancers and compared with clinical findings and *ERBB2 *mRNA expression. Effects of exogenous LL-37 and transgenic overexpression of hCAP18 on ErbB2 signalling were investigated by immunoblotting using extracts from breast cancer cell lines ZR75-1 and derivatives of MCF7. We further analysed the impact of hCAP18/LL-37 on the morphology of breast cancer cells grown in soft agar, on cell migration and on tumour development in severe combined immunodeficiency (SCID) mice.

**Results:**

The expression of *hCAP18 *correlated closely with that of *ERBB2 *and with the presence of lymph node metastases in oestrogen receptor-positive tumours. hCAP18/LL-37 amplified Heregulin-induced mitogen-activated protein kinase (MAPK) signalling through ErbB2, identifying a functional association between hCAP18/LL-37 and ErbB2 in breast cancer. Treatment with LL-37 peptide significantly stimulated the migration of breast cancer cells and their colonies acquired a dispersed morphology indicative of increased metastatic potential. A truncated version of LL-37 competitively inhibited LL-37 induced MAPK phosphorylation and significantly reduced the number of altered cancer cell colonies induced by LL-37 as well as suppressed their migration. Transgenic overexpression of hCAP18 in a low malignant breast cancer cell line promoted the development of metastases in SCID mice, and analysis of hCAP18 transgenic tumours showed enhanced activation of MAPK signalling.

**Conclusions:**

Our results provide evidence that hCAP18/LL-37 contributes to breast cancer metastasis.

## Introduction

The human cathelicidin antimicrobial protein hCAP18 is the single human member of the mammalian cathelicidin family of proteins [1]. The holoprotein consists of a conserved prodomain, cathelin and the non-conserved C-terminal peptide LL-37, which is enzymatically cleaved after secretion [2-4].

Consistent with a role in the first line of defense, hCAP18/LL-37 is widely expressed in leucocytes and in epithelial cells [5,6]. Although initially identified purely as an antimicrobial protein, hCAP18/LL-37 is multifunctional with diverse and significant effects on eukaryotic cells. Thus, LL-37 transactivates the epidermal growth factor receptor (EGFR) inducing cytokine release and cell migration [7,8] and stimulates chemotaxis and angiogenesis through the G-protein coupled receptor, the formyl peptide receptor like-1 (FPRL-1) [9-11]. In line with these findings, current research indicates that hCAP18/LL-37 is actively involved in tissue repair and wound healing [12,13] processes that share fundamental biological features with tumour growth and progression [14].

Antimicrobial proteins, including hCAP18/LL-37, have primarily been proposed as potential anti-tumour agents based on their cytotoxic effects at high concentration [15,16]. However, in a previous study comprising 28 breast cancer samples, we reported that hCAP18/LL-37 was upregulated in breast cancer cells with a correlation between hCAP18 protein levels and tumour grade, whereas in normal mammary tissue it was produced at a low level [17]. We also found that treatment with LL-37 peptide stimulated the proliferation of epithelial cells suggesting that LL-37 may act as a growth factor. Recent findings in lung and ovarian cancer show that overexpression of hCAP18/LL-37 also occurs in other cancer forms and may promote tumour growth [18,19].

Based on our previous study, we were prompted to further explore the role of hCAP18/LL-37 in breast cancer. Here we report the coexpression of *hCAP18 *and *ERBB2 *in breast tumours and their functional cooperation *in vitro *and in a mouse model.

## Materials and methods

### Patients and samples

Breast cancer samples were collected consecutively from patients treated at Danderyd's Hospital, Stockholm, Sweden between 1994 and 1998 (n = 145). Thirty-six samples were excluded due to lack of information about oestrogen receptor (ER) status, lymph node status and/or RNA. The remaining 109 tumours were scored following established guidelines, and ER status was assessed on routinely processed paraffin sections. Healthy breast tissue was obtained from patients undergoing reconstructive surgery (n = 4). The study was approved by the regional committees of ethics and informed consent was obtained from patients and controls.

### Expression analysis of tumour RNA

RNA from human breast cancers was extracted with Trizol (Invitrogen, Paisley, UK) and from mouse tumours with a column-based extraction kit (Bio-Rad Laboratories, Hercules, CA, USA). Random primed reverse transcription and real-time PCR (RT-PCR) analysis for *hCAP18 *were performed as previously described, using 18S RNA for normalisation [17]. *ERBB2 *transcription was quantified using an Assay-on-Demand mixture (Applied Biosystems, Foster City, CA, USA).

### Statistical analyses

Levels of *hCAP18 *were compared with respect to lymph node and ER status by means of analysis of variance (ANOVA). Pearson correlation coefficients were used to assess the association between *hCAP18 *and *ERBB2*. All analyses were performed on logarithmically transformed data using the Statistical Analysis System package, version 9.1 (SAS Institute Inc, Cary, NC, USA). For quantitative evaluation of cell colonies grown in agar, experiments using eight plates for each condition were arranged in a factorial design and the resulting data analysed by ANOVA with the significance level set to α = 0.05. For statistical evaluation of cell migration and mouse tumours and the occurrence of metastases, the non-parametric Mann-Whitney test was used.

### Cell lines

ZR75-1 and MCF7 were obtained from ATCC (via LGC Promochem, Boras, Sweden). The cell line MJ1105 [20], derived from MCF7 cells, was kindly provided by Mikala Egeblad, UCLA, San Francisco, USA. Cells were stably transfected with a vector for bicystronic expression of enhanced green fluorescent protein (eGFP) and hCAP18 (cell line named MJ1105-hCAP) or with the empty eGFP-control (cell line named MJ1105-eGFP), selected by growing in OptiMEM supplemented with 10% FCS and G418 (400 μg/ml), enriched by fluorescence-activated cell sorting (FACS) selection for eGFP expression and maintained as described [17].

### Synthetic peptides

LL-37, LL-25 and scrambled peptide were synthesised and purified by HPLC to a purity of 98% (Polypeptide Laboratories Hilleröd, Denmark, and GeneCust, Dudelange, Luxembourg). The biological activity of LL-37 was confirmed in an antibacterial assay (not shown).

### Mitogen-activated protein kinase activation assay

All experiments were performed on at least two independent occasions. ZR75-1 cells or MJ1105 derivatives were plated in 12-well plates at 100,000 cells per well, and starved for 48 hours in DMEM without FCS. Cells were stimulated with LL-37 and/or Heregulin β3 (HRG) recombinant protein (Upstate, Lake Placid, NY, USA). HRG was used at 2 ng/ml to optimise detection of synergism between HRG and LL-37. The inhibitors bisindolylmaleimide I, PD153035, GM6001, H89, PP2 (all Calbiochem, San Diego, CA, USA), N-acetylcystein, Tiron and pertussis toxin (all Sigma, St Louis, MO, USA), and WRW4 (Phoenix Pharmaceuticals, Belmont, CA, USA) were applied at the indicated concentrations 30 minutes before stimulation with LL-37 and/or HRG. Cells were washed 20 minutes after stimulation with ice-cold PBS containing 1 mM sodium fluoride, 100 μM sodium orthovanadate and 2 mM phenylmethylsulphonylfluoride, and immediately lysed with sodium dodecylsulfate lysis buffer containing the respective inhibitor plus 50 mM dithiothreitol.

### Protein detection

Western blot analysis was essentially performed as described [13], using antibodies against phosphorylated and total protein mitogen-activated protein kinase (MAPK) (Cell Signaling Technology, Beverly, LA, USA), phosphorylated and total protein ERBB2 (Upstate, Lake Placid. NY, USA), and hCAP18 [13], all at 1/2000 dilution. Enhanced chemiluminescence signals (Amersham Biosciences, Piscataway, NJ, USA) were captured by a charge-coupled device camera (Fujifilm, Tokyo, Japan) using Image Gauge (Fujifilm, Tokyo, Japan) for evaluation. Linearity of the signal within the range of our experiments was confirmed using a serial dilution. For normalisation, the Ponceau staining of the blot was scanned and the digitised picture evaluated with the same software. The linear portion of the signal was determined to occur between 5 and 100 μg of total protein extract, therefore about 20 μg was routinely applied.

Immunohistochemistry on cryostat sections from snap-frozen tumours was performed as described previously [13] with affinity purified rabbit anti-LL-37 antibodies at 1/6000 dilution. The presence of breast cancer cells in all tumours and metastases was verified by immunohistochemistry with anti-human leucocyte antigen (HLA) A/C antibodies (Serotec, Raleigh, NC, USA) at 1/40,000 dilution.

### Colony formation assay

One thousand cells (MJ 1105 or ZR75-1) were suspended in 2 ml of 0.35% melted agar in OptiMEM medium (Invitrogen, Carlsbad, CA. USA) with 5% FCS plus LL-37/HRG, and plated in 50 mm gridded dishes containing a solidified layer of 0.7% agar in the same medium. After 12 days, the top layer of the culture was stained with 0.2% p-iodonitrotetrazolium violet (Sigma, St Louis, MO, USA). Colonies within a grid of 10 × 10 that were larger than 100 μM in diameter (about 200 colonies/plate) were counted and their morphology categorised. All evaluations were performed in a blinded manner. The experiments were independently performed three times.

### Cell migration assays

The Boyden chamber migration assays were carried out in the presence or absence of LL-37 and/or LL-25. Briefly, cells were serum starved for four hours in 0.2% BSA containing α-MEM medium. Cells were then trypsinised, harvested and resuspended in serum-free α-MEM medium. Chemotactic activity was determined in a Boyden chamber system (Neuroprobe Inc, Gaithersburg, MD., USA) with a 8 μm pore size polycarbonate membrane (Neuroprobe Inc, Gaithersburg, MD., USA) separating the two chambers. Serum-free α-MEM alone or with LL-37 and/or LL-25 was added to the lower chamber with final concentrations of 2 μM and 1 μM, respectively. Cells were loaded to the upper chamber at a concentration of 5 × 10^4 ^cells in 500 μl of serum-free α-MEM. Chambers were incubated at 37°C for five hours in a 95% air, 5% carbon dioxide atmosphere. The upper medium was then removed, the chamber was disassembled and membranes were fixed in 96% methanol for two minutes. The membranes were rinsed in distilled water and subsequently stained in Giemsa for two minutes, followed by two washing steps. Then, the cells on the upper side of the filters were cleaned off. Cells in the filter were counted under a microscope at a magnification of 20. To allow for comparison between multiple assays, the data were normalised and expressed as migration rate of the cells compared with the control chamber.

### Tumorigenicity studies in SCID mice

The study was approved by the Animal Assurance Agency R5002-01 (ethical permits N421/04 and N235/05). MJ1105-hCAP18 and MJ1105-eGFP cells were trypsinised and suspended in PBS/1 mM magnesium chloride at 50 million/ml. Ten million cells (200 μl) were injected subcutaneously into the mouse. These cells require oestrogen for tumour formation, so slow-release 17-β oestradiol pellets (1.7 mg total dose; Innovative Research of America, Sarasota, FL, USA) were implanted the day before tumour cell injection. The test group injected with the hCAP18 transgenic cell line and the group injected with the control cell line each consisted of five mice. Mice were observed daily and palpated for tumour formation twice a week. Mice were sacrificed when the tumour reached about 1 cm^3 ^in size. Tumours were excised and snap frozen, and used for immunohistochemistry, protein and RNA extraction as described. For detection of metastasising cells, spleen and liver were disaggregated to single cell suspensions, and after lysis of erythrocytes with 0.17% ammonium chloride and removal of debris by centrifugation, analysed for the presence of eGFP expressing cells by FACS analysis.

## Results

### hCAP18 expression correlates with the expression of ERBB2 and is associated with lymph node metastasis in estrogen receptor positive human breast cancer

**Figure 1:**
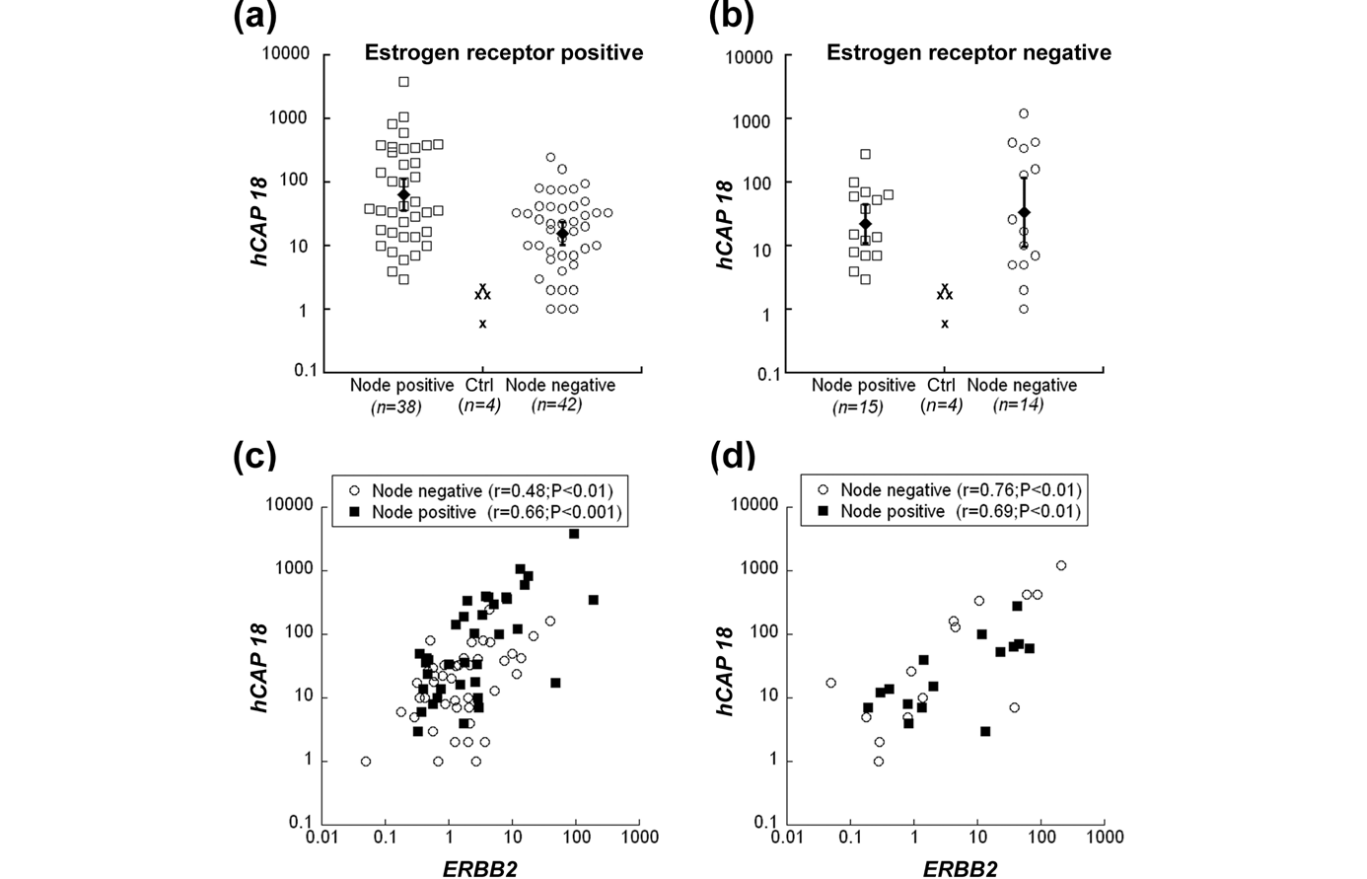
*hCAP18 *transcript levels in relation to *ERBB2 *levels, lymph node status and oestrogen receptor (ER) status. All transcription levels determined by real-time reverse transcriptase PCR are displayed relative to the mean of four control samples. **(a) **Significantly higher levels of *hCAP18 *transcript for lymph node positive compared with lymph node-negative ER-positive patients (p < 0.001). **(b) **No significant difference in *hCAP18 *levels with respect to lymph node status was observed for ER-negative patients (a, b) filled markers and error bars represent geometric means and 95% confidence intervals. The association between *hCAP18 *levels and lymph node status was significantly different for (a) ER-positive and (b) ER-negative patients (p = 0.01). **(c, d) **Significant correlation between levels of *hCAP18 *and *ERBB2 *was observed in all groups, whether (c) ER positive or (d) ER negative, and irrespective of lymph node status (squares versus circles).

Previously we have shown that *hCAP18 *mRNA and protein are overexpressed in human breast cancer samples [17]. To explore its possible association with tumour development, we investigated *hCAP18 *mRNA levels in an extended panel of human breast cancer samples (n = 109). Results of quantitative RT-PCR demonstrated that the average level of *hCAP18 *mRNA expression was at least one order of magnitude higher in breast cancer tissues in comparison with normal breast tissue (Figure 1a,b). Of note, only eight out of the 109 tumours expressed *hCAP18 *within the low range of control samples and none of these tumours had lymph node metastases at the time of surgery [see additional data file 1]. Stratifying the patient material based on ER expression, and the presence of lymph node metastasis we found that *hCAP18 *expression was significantly higher (p < 0.001) in ER-positive tumours (n = 38) with lymph node metastases than in tumours without lymph node metastasis (n = 42) associating the expression of *hCAP18 *with metastasis formation in breast cancer (Figure 1a). Although the expression of *hCAP18 *was significantly greater in ER-negative tumours compared with control tissues, there was no clear association with lymph node metastasis at the time of primary surgery (Figure 1b). However, our results indicate that high *hCAP18 *expression is associated with lymph node metastasis at least in ER-positive human breast cancer.

Amplification and overexpression of the tyrosine kinase receptor gene *ERBB2 *is a hallmark of metastatic development in breast cancer [21]. Because LL-37 has been linked to EGFR signalling [7,8], we investigated if increased *hCAP18 *expression was associated with changes in *ERBB2 *levels. Our data demonstrate a highly significant correlation between the expression of both genes in ER-positive as well as in ER-negative tumours (Figure 1c,d). Neither *ERBB2 *nor *hCAP18 *transcription levels in breast cancer patients correlated with relapse or mortality after 5 to 10 years of follow-up (data not shown).

### hCAP18 and ErbB2 are functionally connected in breast cancer cells

**Figure 2:**
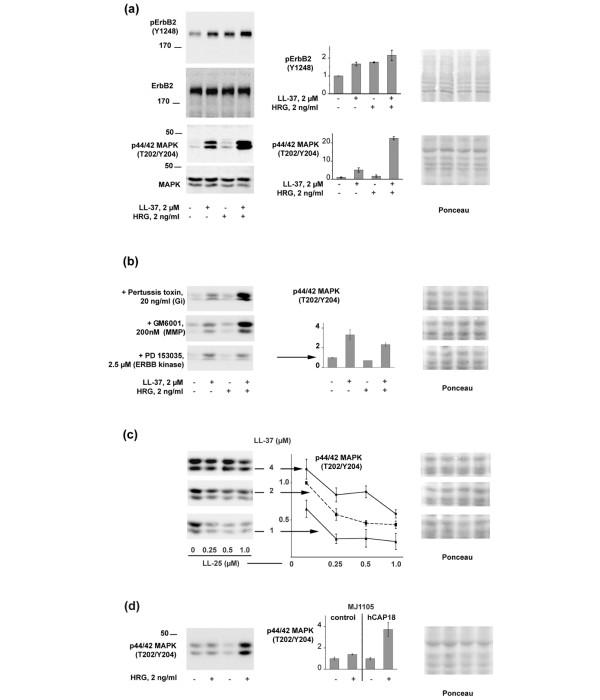
LL-37 synergistically enhances Heregulin-induced mitogen-activated protein kinase (MAPK) phosphorylation through the ERBB2 receptor. **(a) **Phosphorylation of ERBB2 (upper panel) and MAPK (lower panel) by treatment of ZR75-1 cells with LL-37 and Heregulin β3 (HRG) on their own and in combination. The diagram shows the mean phosphorylation level as evaluated by Western blot analysis, normalised against Ponceau staining and relative to untreated samples. On the left side is shown one representative of the triplicates used for evaluation. The lower rows show Western blots against total ERBB2 and MAPK, demonstrating an unaltered protein level, and Ponceau staining of the corresponding sections of the blot. **(b) **Inhibition of MAPK activation in ZR75-1 by the ERBB inhibitor PD153035 but not by pertussis toxin or metalloprotease inhibitor GM6001. The arrow points to the histogram for PD153035 treatment. **(c)** LL-25 inhibits the LL-37 dependent activation of MAPK. Experiments were performed in the MJ1105 control cell line using HRG at 2 ng/ml, and by varying the concentrations of both LL-37 and LL-25 as displayed. The diagram shows the levels of MAPK phosphorylation relative to the conditions of 2 μM LL-37.** (d)** Synergistic effect between HRG and endogenous production of transgenic hCAP18 in the MCF-7 derivative MJ1105. For all experiments, the Ponceau staining used for normalisation is shown. All conditions were run in triplicates (n = 3), and in addition repeated at least on one independent occasion.

To investigate whether hCAP18 regulates the expression of *ERBB2*, we established a transgenic cell line derivative from a low malignant ER-positive breast cancer line, MJ1105 [22], which has no amplification of *ERBB2 *and expresses hCAP18 at a low levels similar to those of normal mammary tissue. The low expression of ERBB2 remained unchanged [see additional data file 2], so we further investigated whether LL-37 had a functional influence on ERBB2. And because LL-37 is reported to induce phosphorylation of MAPK via the EGFR [7], we assessed its effect on signaling through ErbB2 using recombinant HRG as positive control. The experiments were carried out in MJ1105 and in the ER-positive breast cancer line ZR75-1 [22]. Both LL-37 and HRG induced phosphorylation of ERBB2 and MAPK in both cell lines (shown in Figure 2 for ZR75-1). When LL-37 and HRG were added together, a synergistic increase in MAPK phosphorylation was observed. Immunoblotting showed that the amount of ERBB2 and ERK 1/2 protein remained unchanged, indicating that LL-37 and HGR did not affect their expression, only their activation (Figure 2a).

A scrambled version of LL-37 [23] was without activity (not shown), confirming the specificity of LL-37 in our experiments. For LL-37, a micromolar concentration was required to achieve significant synergistic activity (data not shown). This concentration range of LL-37 was previously demonstrated to increase proliferation and migration of epithelial cells and to induce angiogenesis [8,17,23].

PD153035, an inhibitor of tyrosine kinase activity of the ERBB family, blocked the HRG dependent MAPK activation (Figure 2b) at 2.5 μM. The activation of MAPK induced by LL-37 on its own was maintained, but the synergistic effect seen with both substrates was lost in the presence of PD153035 (Figure 2b). At 20 nM, a concentration sufficient to completely block the EGFR [24], PD153035 only caused a slight inhibition. Thus the EGFR does not seem to contribute significantly to the observed effect.

LL-37 has been shown to activate cell signalling through pertussis toxin sensitive G proteins [8,10,23]. The activation of EGFR by LL-37 was previously demonstrated to involve the release of heparin-bound EGF by metalloproteases that were blocked with the inhibitor GM6001 [7]. Neither of these alternatives, however, seemed responsible for our findings, since the effect of LL-37 both in absence and presence of HRG was unaffected by pertussis toxin or GM6001 (Figure 2b). We also used the WRW4, an antagonist of LL-37 at the G-protein coupled receptor FPRL1 [25], and inhibitors against PKA, PKC and c-src, which can perform a crosstalk between ERBB2 and a G-protein coupled receptor [26]. In addition we tested inhibitors against reactive oxygen species, which can influence tumourigenicity by activating EGFR-related pathways [27]. The synergistic effect between LL-37 and HRG on the level of activated MAPK was unaffected by these treatments [see additional data file 3].

LL-25, a synthetic derivative of LL-37, lacking the 12 C-terminal amino acids, had a minimally stimulatory effect on the phosphorylation of MAPK on its own (not shown), but strongly inhibited the effects induced by LL-37 (Figure 2c). The inhibition was dependent on the LL-37/LL-25 molar ratio, and not on the absolute concentration of LL-25, suggesting that LL-25 serves as a competitive inhibitor of LL-37. A scrambled version of LL-37 [23] was without activity (not shown), confirming the specificity of LL-37 in our experiments.

MJ1105 cells showed a similar behaviour in this assay. However, the response to HRG was strongly elevated when hCAP18 was overexpressed from a transgene, indicating that transgenic expression partially replaced the exogenous addition from LL-37 (Figure 2d).

### LL-37 alters anchorage independent growth morphology of breast cancer cells

**Figure 3:**
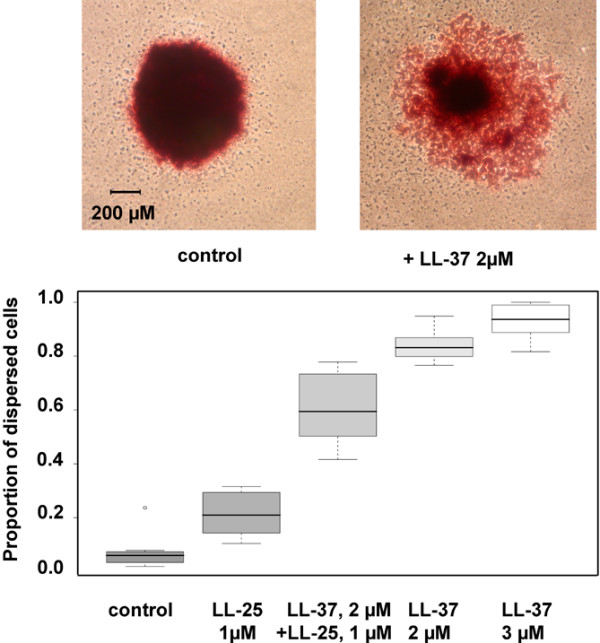
LL-37 induces morphological changes in MJ1105 soft agar clones. The upper row shows an example of a colony in absence and in presence of LL-37. For the quantitative evaluation displayed in the diagram, cell culture experiments for each condition (n = 8) were arranged in a factorial design and the resulting data analysed by analysis of variance (ANOVA), the significance level set to α = 0.05. The proportion of cell colonies with LL-37 induced morphological alterations was significantly lower in presence of LL-25 (p < 0.01). The experiment was repeated on three independent occasions.

To investigate whether LL-37 influences tumour cell behaviour, we studied the effect of LL-37 on colony formation of MJ1105, with or without transgenic *hCAP18 *expression, and ZR75-1 in soft agar. LL-37 in the presence or absence of HRG did not significantly affect the number of colonies, but profoundly affected their morphology irrespective of the cell line. In the presence of LL-37, colonies became less compact and were surrounded by satellites (shown for MJ1105 in Figure 3). This observation shows that LL-37 impacts the growth pattern of breast cancer cells and suggests that LL-37 promotes a migratory cell phenotype. The addition of 1 μM LL-25, which was sufficient to substantially inhibit the LL-37-induced MAPK phosphorylation, significantly reduced the number of dispersed colonies (Figure 3). The three cell lines behaved similarly [see additional data file 4]. Thus, the LL-25 peptide inhibited not only MAPK activity induced by LL-37, but also the soft agar growth morphology of breast cancer cells.

### LL-37 stimulates the migration of breast cancer cells *in vitro*

**Figure 4:**
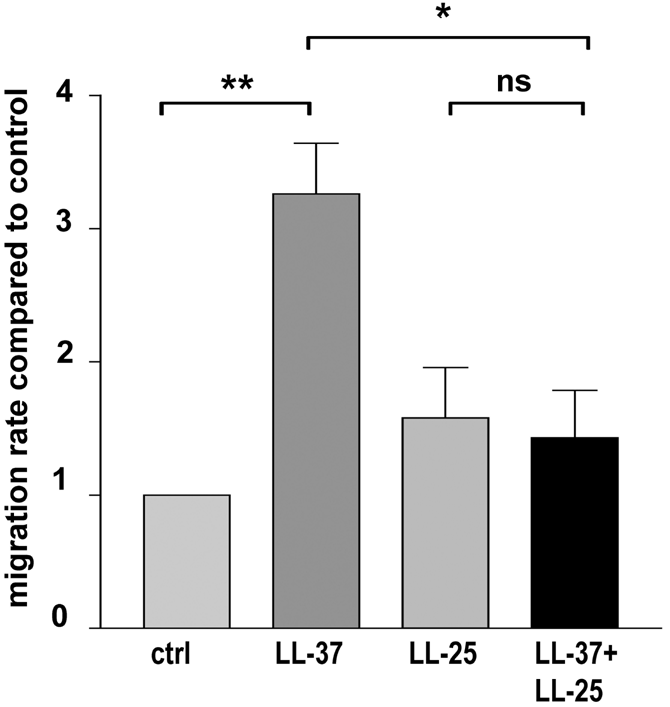
LL-37 at 2 μM increases the migration of MCF-7 breast cancer cells in a Boyden Chamber assay. The stimulatory effect induced by LL-37 was abrogated when LL-25 at 1 μM was added together with LL-37 in the lower chamber. Control experiments were with medium only in the lower chamber, or with LL-25 in the medium, respectively. The results are given as mean ± standard error of the mean, n = 4. * p < 0.05, ** p < 0.01, ns = non significant.

The change in cell colony phenotype was suggestive of an influence of LL-37 on cell migration, so we evaluated the effect of LL-37 on MCF7 breast cancer cell migration using a Boyden chamber assay. In this assay, the presence of 2 μM LL-37 tripled the number of migrating MCF7 cells (p < 0.001), compared with controls. In accordance with our *in vivo *data, the presence of LL-25 (1 μM) abolished the migratory effect of LL37 (p < 0.01), thus confirming its inhibitory potential, although on its own LL-25 did not have a significant effect on cell migration (Figure 4).

### Overexpression of hCAP18 in breast cancer cells enhances metastasis formation in SCID mice

**Figure 5:**
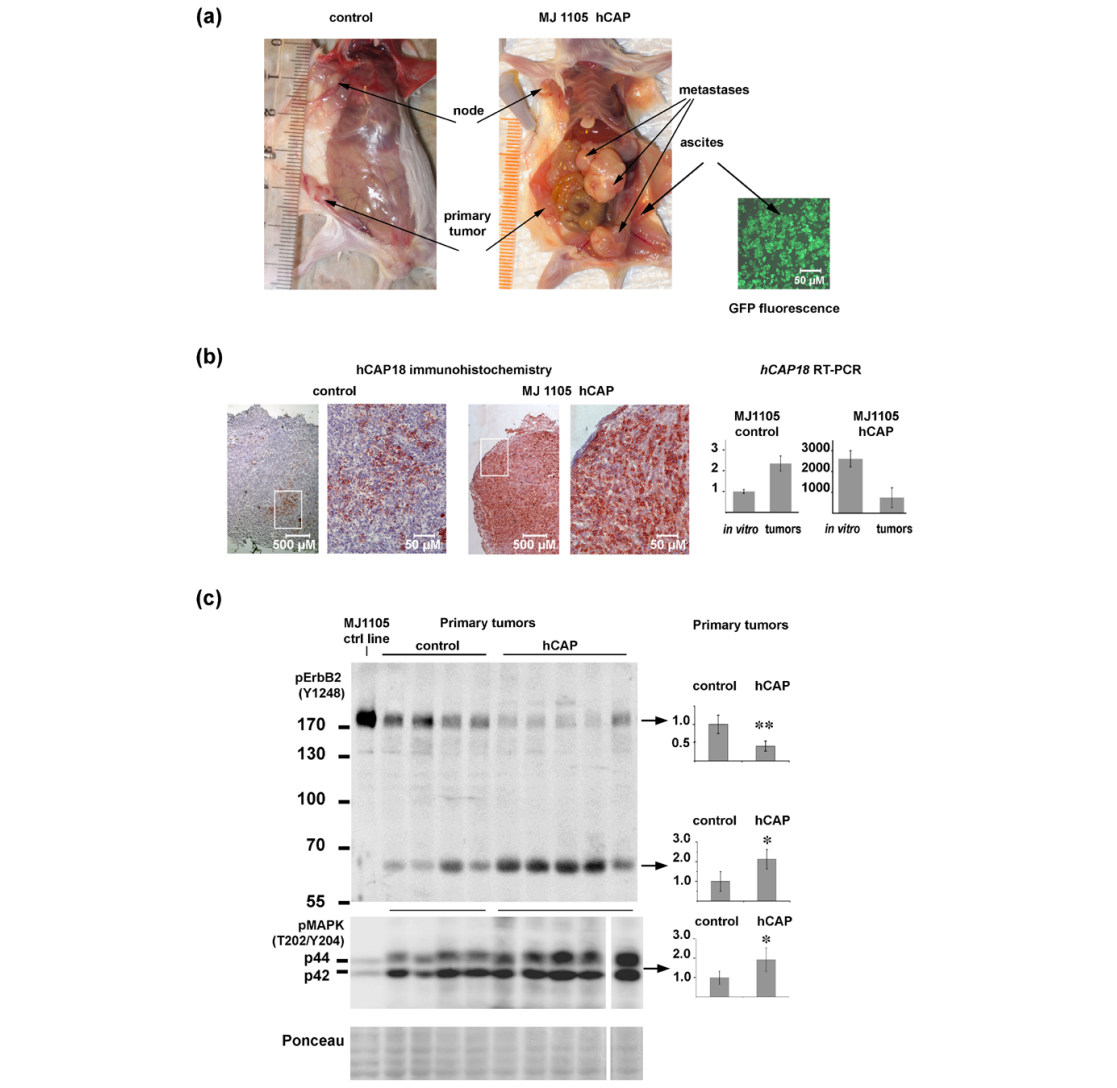
Overexpression of *hCAP18 *in MJ1105 cells increases metastasis formation in severe combined immunodeficiency (SCID) mice. **(a) **Metastasis formation displayed in a mouse injected with MJ1105-hCAP18 cells compared with control mouse treated with MJ1105 lacking hCAP18. The right panel shows tumour cells from ascites fluid detected by fluorescent transgene-coupled enhanced green fluorescent protein (eGFP) expression. **(b) **Expression analysis of hCAP18/L-37 in mouse tumours. (Left) Immunohistochemistry with anti-LL-37 antibodies demonstrating strong expression in transgenic tumours, and minor local induction in control tumours. Right, the expression of *hCAP18 *mRNA determined by reverse transcriptase PCR in tumours and transgenic cell lines. All comparisons are made to the expression of MJ1105 control cell line, which is set to one.** (c)** Western blot analysis demonstrates increased mitogen-activated protein kinase (MAPK) phosphorylation and increased degradation of phosphorylated ERBB2, in *hCAP18 *transgenic primary tumours compared with control primary tumours. On the left side of the blot, the control cell line is shown for comparison. Diagrams to the right illustrate the expression levels for respective tumour group, their statistic significance (two-tailed t-test, equal variance) indicated by asterix.

To extend our *in vitro *findings to *in vivo *tumour growth and metastasis, we investigated the effect of hCAP18/LL-37 in a xenograft model. To this end, we established primary tumours with *hCAP18 *transgenic and control derivatives of MJ1105 cells in severe combined immunodeficiency (SCID) mice and monitored tumour growth and metastasis formation (Table 1 and Figure 5). As determined by RT-PCR, the transgenic cell line expressed hCAP18 at the level of high expressing breast tumours, whereas the expression of the control cell line was at the level of unaffected breast tissue (Figure 5b).

**Table 1:** Survey of mouse tumours derived from *hCAP18 *overexpressing MJ1105 and its control.

**Cell line**	**Mouse**	**Sacrificed day past inoculation**	**Primary tumour size (mm)**	**Metastases**	**Lymph** **nodes**	**Ascites with eGFP cells**	**eGFP cells in spleen**	**GFP cells in liver**
**MJ 1105-** **hCAP18**	h1	43	6 × 6 × 6					
	h2	71	10 × 8 × 8				+	+
	h3	71	8 × 8 × 8		3			+
	h4	75	8 × 8 × 8	3 abdominal	2	+		
	h5	90	8 × 5 × 4		1			
								
**MJ1105** **eGFP**	I1	43	10 × 10 × 10					
	I2	43	8 × 7 × 6					
	I3	90	7 × 10 × 6					
	I4	90	8 × 4 × 4		1			

For statistical evaluation, each visible metastasis or node, or the presence of fluorescent cells in ascites/spleen or liver, are counted as one event each. Using the Mann-Whitney U-test, the median number of metastatic events between the groups is significant (p = 0.05). eGFP = enhanced green fluorescent protein.

*In vitro*, the colony formation assay did not show any distinguishable features for the transgenic lines [see additional data file 4]. In the mouse, however, we observed a significant difference between transgenic and control cells in metastasis formation (Table 1.) Four out of five mice injected with the hCAP18-overexpressing breast cancer cells developed metastases, three of them in multiple forms and/or loci (Table 1). Metastases were detected in lymph nodes, abdominal tumours, ascites fluid and EGFP-expressing MJ1105-hCAP18 cells in spleen and liver (Figure 5a and Table 1). In contrast, only one lymph node metastasis was detected in one of the mice injected with the control cells (Table 1), and this was located on the same side as the primary tumour. The presence of human breast cancer cells in all tumours and metastases was verified by detecting the expression of eGFP fluorescent marker in the ascites fluid (Figure 5a), and by immunohistochemistry with anti-HLA-ABC antibodies (not shown) on the solid tumours.

High expression of *hCAP18 *in all primary and secondary tumours of test mice injected with MJ1105 hCAP was verified by RT-PCR and confirmed by immunohistochemistry (Figure 5b and additional data file 5). Some tumour regions stained weakly, even with HLA staining (not shown), indicating infiltration by mouse cells. Interestingly, in primary tumours from the control cell line, RT-PCR analysis of *hCAP18 *mRNA showed a two-fold elevation compared with the expression in cell culture (Figure 5b). Immunohistochemistry revealed that hCAP18 was produced in small foci within all primary control tumours, thus confirming that spontaneous, local upregulation of hCAP18 occurred in these tumours *in vivo *(Figure 5b). No alteration of the *ERBB2 *transcription level, compared with the parental cell lines, was detected in control or test samples [see additional data file 2].

Western blot analysis revealed significant differences between primary tumours in test and control mice. The level of phosphorylated MAPK was higher (p < 0.05) in *hCAP18 *transgenic tumours compared with the control group. In the transgenic tumours, a decrease of phosphorylated p185 ERBB2 was found (Figure 5c) whereas a band of 65 kDa was increased in intensity, indicative of ERBB2 degradation. An additional blot using antibodies against total ERBB2 showed the same image and thus confirmed that the bands indeed were derived from ERBB2 (not shown).

## Discussion

Expanding from our previous findings, we demonstrate that *hCAP18 *is highly expressed in breast cancer. Only few tumours expressed *hCAP18 *mRNA within the range of control samples, and none of these showed evidence of metastases. Stratifying the material we found that in the ER-positive tumours, but not in ER-negative tumours, the level of hCAP18 expression was significantly higher in patients with lymph node metastasis. ER positivity is reported to be associated with more benign disease, so our findings may seem contradictory. However, breast cancer biology and heterogeneity remain insufficiently understood and still today, no single marker has been identified that will consistently predict prognosis. In fact, microarray studies have demonstrated that ER-positive tumours are a highly heterogeneous group, and that the overall favourable prognosis attributed to ER-positive status proved valid for a subgroup of tumours, whereas another group of ER-positive tumours was associated with poor outcome [28].

Of particular interest in this context is the recent identification of a 'wound response signature' that from established biomarkers such as tumour grade, lymph node status and ER-status, was shown to independently predict prognosis in breast cancer with significantly higher accuracy than established risk factors [29]. Specifically, it was shown that the expression of such a wound signature in breast tumours significantly affected metastasis potential and reduced survival. Considering that LL-37 is emerging as an integral part of the innate reaction to wounding, being rapidly and strongly upregulated in response to injury and also involved in the healing process, it can be hypothesised that similar cellular programs are at play in cancer progression.

Gene amplification and protein overexpression of the tyrosine kinase receptor ErbB2 is considered a hallmark of metastatic development and poor outcome in breast cancer [21]. Our data demonstrate a significant correlation between the transcription levels of *hCAP18 *and *ERBB2 *genes in ER-positive as well as in ER-negative tumours. Despite this, there was no correlation for *hCAP18 *or *ERBB2 *expression and survival at five-year follow-up in any of the groups, again underscoring the limitations using single transcription markers in predicting disease outcome.

The control breast cancer cells used for the mouse study have only marginal expression of hCAP18 when grown *in vitro*. However, after forming primary tumours at the site of cell injection, we detected a focal upregulation of hCAP18 mRNA and protein in all mouse control tumours (Figure 5b). This phenomenon supports the notion that upregulation of hCAP18 is a common event during breast cancer development, in agreement with our findings in the clinical samples. Recently LL-37 was shown to stabilise the Hypoxia-Inducible factor alpha, and consequently upregulate vascular endothelial growth factor, in human keratinocytes [30], thus linking LL-37 to hypoxia similar to its porcine counterpart PR39 [31]. Thus, one can hypothesise that hypoxia may be the biological basis for the upregulation of hCAP18/LL-37 that we observed in the mouse tumours.

Although our studies show that hCAP18/LL-37 synergistically enhances ERBB kinase signalling, the mechanism remains unknown. By the use of specific inhibitors (Figure 2b), we excluded the previously reported mechanisms of LL-37, that is the release of EGF-like factors through activation of metalloproteases and/or pertussis toxin sensitive activation of G proteins. Based on similar studies on lung cancer cells [19] and in line with structural studies on LL-37 [32], it was suggested that LL-37 causes its effects by electrostatic interactions with the cell membrane rather than with a receptor. In our study, we have identified a truncated N-terminal peptide, LL-25, that acts as a potent inhibitor of both LL-37 signalling and of LL-37-induced migration and alteration of cancer cell colony morphology. We find it difficult to understand how a fragment of LL-37 would inhibit membrane interactions, and favour the hypothesis that LL-37 interacts with ERBB kinases via a yet unidentified receptor. Still, a direct effect of LL-37 on the ERBB receptors cannot be excluded. Whatever the mechanism, the fact that the effects of LL-37 can be inhibited opens up the possibility of therapeutic targeting.

The results from the colony formation and migration assays confirm that LL-37 expression contributes to metastases, as hypothesised on the basis of our findings in the clinical samples. The soft agar growth pattern is indicative of cellular behaviour and the profound phenotypic changes induced by LL-37 peptide treatment, most likely reflect an enhanced migratory and invasive capacity in these cells. The colony morphology induced by LL-37 was strikingly similar to the growth pattern reported for a melanoma cell line exposed to EGF-like peptides [33], and for mammary epithelial cells overexpressing ERBB2 [34], which was hypothesised to mirror an increased metastatic potential [34]. The 2 μM concentration of LL-37 used in this experiment is well below cytotoxic levels and was previously shown to stimulate cell proliferation and migration [17,18]. In contrast, to what was recently reported for lung cancer cells [19], we did not detect significant differences in the number of colonies after treatment with LL-37 which may be due to underlying biological differences between the cancer cell types utilised in these experiments. It should be highlighted that our analysis on breast tumour samples is presently the only one to show a correlation of *hCAP18 *expression with a particular tumour cell phenotype, still pending study in other tumour types.

In accordance with the findings in the clinical studies and in the *in vitro *experiments, we found a significant increase in metastases in the SCID mice injected with the *hCAP18 *transgenic cell line. Not only did we detect lymph node metastases but also distant metastases indicative of lymphatic as well as haematic spread of tumour cells. The control breast cancer cells used for the mouse study have only marginal expression of hCAP18 when grown *in vitro*. However, after forming primary tumours at the site of cell injection, we detected a focal upregulation of hCAP18 mRNA and protein in all mouse control tumours indicating that local upregulation of hCAP18 occurred when these tumours formed *in vivo *(Figure 5b). This phenomenon supports the notion that upregulation of hCAP18 is a common event during breast cancer development, in agreement with our findings in the clinical samples.

Western blot analysis of the mouse tumours confirmed that the MAPK activation by hCAP18/LL-37 that we observed *in vitro *reflects events of *in vivo *tumourigenesis. The partial degradation of ERBB2 in overexpressing tumours is worthy of note. A reduced amount of intact protein, at a constant transcription rate, is characteristic of receptor degradation after activation and internalisation, as reported for ERBB2 following long-term ligand activation [35]. Truncated versions of ERBB2 have been shown to actively participate in signalling [36] and to be resistant to trastuzumab treatment in breast cancer [37].

We did not detect the presence of truncated ERBB2 in our *MAPK *activation assays, which may reflect that the activation time might have been too short to induce degradation. However, the cellular environment should also be considered: in the mouse the extracellular proteins or domains on the tumour cell surface are exposed to proteases from the surrounding stroma, which might contribute to their degradation, or in the case of hCAP18, its activation. This would also explain why transgenic *hCAP18 *did not appear to alter the properties in the colony formation assay, but still induced metastasis formation in the mouse. In correlation with this hypothesis, recent studies show that the expression of metalloprotease MMP9 from the tumour surrounding stroma, unlike its expression in tumour cell cytoplasm, correlates with unfavourable prognosis in ERBB2 positive breast cancer [38].

The correlation between hCAP18 and ERBB2 expression that was observed in the human tumours did not occur *in vitro *or in the mouse model. We favour the hypothesis that the upregulation of both occurs independently, but due to their functional collaboration is selected for during the evolution of human breast cancer.

The role for hCAP18/LL-37 as a proinflammatory molecule in innate immunity is well established. In this context, LL-37 alerts the immune system in case of injury and microbial invasion. It is conceivable that broad defense strategies involving hCAP18/LL-37 evolved to provide sustained protection and prompt repair, aiming to fully restore tissue integrity. This would be consistent with our recent understanding of hCAP18/LL-37 as a stimulant of cell migration and proliferation and as a promoter of wound healing [12,13]. Thus, in view of the emerging link between chronic inflammation and cancer [14], hCAP18/LL-37 may contribute to cancer as a novel and versatile player, not only through its proinflammatory actions but also through its growth-factor like properties.

The data presented herein and recent observations in lung and ovarian cancer [18,19], point to a role for hCAP18/LL-37 in cancer progression and spread. The underlying molecular mechanisms, however, remain to be clarified. hCAP18/LL-37 is emerging as a multifunctional molecule progressively implicated in multiple highly significant processes in cancer development. hCAP18/LL-37 may constitute a putative therapeutic target to prevent progression to metastatic disease.

## Conclusions

In summary, we show that hCAP18/LL-37 is highly upregulated in breast cancer correlating with the expression of *ERBB2. In vitro*, we also show that they are functionally connected, in that hCAP18/LL-37 amplifies MAPK signalling through ErbB2 and that treatment with LL-37 peptide alters the growth phenotype and stimulates the migration of breast cancer cells. Finally, overexpressing hCAP18 in a low malignant breast cancer cell line promotes metastatic disease in a SCID mouse model. Taken together, our data expand on recent findings in lung and ovarian cancers and demonstrate a novel role for the single human cathelicidin protein hCAP18/LL-37 in breast cancer.

## Abbreviations

ANOVA: analysis of variance; BSA: bovine serum albumin; DMEM: Dulbecco's modified eagle's media; eGFP: enhanced green fluorescent protein; EGFR: epidermal growth factor receptor; ER: oestrogen receptor; FACS: fluorescence-activated cell sorting; FCS: fetal calf serum; FPRL-1: formyl peptide receptor like-1; hCAP: human cathelicidin antimicrobial protein; HLA: human leucocyte antigen; HPLC: high-performance liquid chromatography; HRG: heregulin β3; MAPK: mitogen-activated protein kinase; PBS: phosphate buffered saline; RT-PCR: reverse-transcription polymerase chain reaction; SCID: severe combined immunodeficiency.

## Competing interests

MS is a founder of Lipopeptide AB, a company that develops pharmaceuticals based on LL-37 for wound healing. GW and MS are inventors of patents identifying LL-37 as a potential target in cancer. GW and MS have transferred the above mentioned patent rights to Karolinska Development which now owns these rights.

## Authors' contributions

MS and GW are responsible for developing the hypothesis and writing the manuscript and GW for planning and executing the experiments. FG performed the statistical analysis of the tumours and their expression. AL and SR collected and evaluated the clinical material. FS supported the analysis of the mouse experiments and performed the statistics for the molecular analysis. CIC performed FACS analysis and supported the setup of the transgenic lines. AP contributed to the critical evaluation and interpretation of data. SZ performed part of the inhibitory studies and characterisation of cell lines. ZS and RM performed the migration assay.

## Supplementary Material

Additional file 1 A MS Word file containing a table that lists the human tumours and cell lines used in this study. The transcription levels of *hCAP18 *and *ERBB2 *as determined by RT-PCR are displayed relative to the mean of the four unaffected breast tissue control samples. n.d. = not done. Click here for file

Additional file 2 A MS Word file containing a table that lists the transcription levels of *hCAP18 *and *ERBB2 *in mouse tumours. The values are relative to the MJ1105 control cell line as determined by RT-PCR, and normalised against total RNA as determined by 18S. Click here for file

Additional file 3 A pdf file containing a figure of a Western blot analysis excluding cross-talk mechanisms for the synergistic effect of LL-37 and HRG on MAPK phosphorylation. (a) The analysis on ZR75-1 cells stimulated with LL-37 and/or HRG after 30 minutes pretreatment with inhibitors as indicated. The concentration of inhibitors is listed together with their prime targets. (b) The evaluation of the effect of WRW4, untreated samples being run side-by-side with treated samples to exclude even minimal differences. All experiments were run in triplicates (n = 3), and repeated at one independent occasion. Click here for file

Additional file 4 A pdf file containing a figure displaying the rate of morphological changes in soft agar clones from ZR75-1, MJ1105 control and *hCAP18 *transgenic cells, as evaluated in Figure 3 (n = 4). Click here for file

Additional file 5 A pdf file containing a figure showing the immunohistochemical analysis with anti-LL-37 antibodies on all mouse tumours in this study. Click here for file
