# Supplementary material for: Human antimicrobial protein hCAP18/LL-37 promotes a metastatic phenotype in breast cancer
Source: Breast Cancer Res. 2009 Jan 30;11(1):R6. doi: 10.1186/bcr2221 (PMC2687709; doi:10.1186/bcr2221)
Supplement: Additional file 1 — A MS Word file containing a table that lists the human tumours and cell lines used in this study. The transcription levels of hCAP18 and ERBB2 as determined by RT-PCR are displayed relative to the mean of the four unaffected breast tissue control samples. n.d. = not done. [file bcr2221-S1.doc]

**Additional data file 1.** Human tumors and cell lines used in this study. The transcription levels of *hCAP18* and *ERBB2* are displayed relative to the mean of the four unaffected breast tissue control samples. n.d.: not done.

| no | age | tumor  size/mm | Elston  grade | qPCR  hCAP18/18S | qPCR  ERBB2/18S | ER/NOD |
| --- | --- | --- | --- | --- | --- | --- |
| 1 | 48 | 8 | 1 | 1 | 2.7 | ER+/N- |
| 2 | 52 | 15 | 3 | 20 | 1.1 | ER+/N- |
| 3 | 70 | 20 | 2 | 17 | 0.3 | ER+/N- |
| 4 | 60 | 60 | 2 | 7 | 2.9 | ER+/N+ |
| 5 | 49 | 15 | 2 | 3 | 13.5 | ER-/N+ |
| 6 | 64 | 22 | 2 | 7 | 2.1 | ER+/N- |
| 7 | 53 | 40 | 3 | 70 | 44.5 | ER-/N+ |
| 8 | 59 | 16 | 2 | 76 | 2.3 | ER+/N- |
| 9 | 76 | 42 | 3 | 10 | 1.4 | ER-/N- |
| 10 | 41 | 12 | 2 | 24 | 11.6 | ER+/N- |
| 11 | 73 | 14 | 2 | 160 | 39.0 | ER+/N- |
| 12 | 61 | 16 | 3 | 7 | 1.4 | ER+/N- |
| 13 | 79 | 24 | 2 | 10 | 2.9 | ER+/N+ |
| 14 | 1 | 38 | 3 | 29 |  | ER+/N+ |
| 15 | 85 | 18 | 3 | 7 | 39.0 | ER-/N- |
| 16 | 63 | 24 | 1 | 8 | 0.6 | ER+/N+ |
| 17 | 52 | 18 | 2 | 33 | 1.4 | ER+/N- |
| 18 | 41 | 40 | 3 | 8 | 0.8 | ER-/N+ |
| 19 | 60 | 28 | 3 | 17 | 48.2 | ER+/N+ |
| 20 | 43 | 24 | 3 | 18 | 2.7 | ER+/N+ |
| 21 | 72 | 35 | 1 | 79 | 3.5 | ER+/N- |
| 22 | 66 | 20 | 1 | 2 | 2.0 | ER+/N- |
| 23 | 54 | 24 | 3 | 12 | 0.3 | ER-/N+ |
| 24 | 44 | 10 | 3 | 13 | 5.3 | ER+/N- |
| 25 | 68 | 11 | 2 | 32 | 1.3 | ER+/N- |
| 26 | 46 | 35 | 3 | 65 | 37.2 | ER-/N+ |
| 27 | 69 | 19 | 2 | 2 | 3.7 | ER+/N- |
| 28 | 72 | 21 | 1 | 338 | 2.0 | ER+/N+ |
| 29 | 48 | 18 | 2 | 8 | 0.9 | ER+/N- |
| 30 | 75 | 18 | 1 | 203 | 3.4 | ER+/N+ |
| 31 | 68 | 7 | 2 | 33 | 0.9 | ER+/N- |
| 32 | 45 | 19 | 2 | 42 | 1.7 | ER+/N- |
| 33 | 30 | 29 | 2 | 2 | 0.3 | ER-/N- |
| 34 | 47 | 100 | 3 | 39 | 1.4 | ER-/N+ |
| 35 | 48 | 15 | 3 | 14 | 0.8 | ER+/N+ |
| 36 | 61 | 7 | 2 | 9 | 1.3 | ER+/N- |
| 37 | 56 | 9 | 1 | 16 | 1.5 | ER+/N+ |
| 38 | 54 | 100 | 2 | 38 | 7.6 | ER+/N- |
| 39 | 73 | 20 | 2 | 142 | 1.3 | ER+/N+ |
| 40 | 70 | 35 |  | 53 | 23.1 | ER-/N+ |
| 41 | 81 | 22 | 3 | 1 | 0.7 | ER+/N- |
| 42 | 44 | 17 | 1 | 34 | 1.0 | ER+/N+ |
| 43 | 39 | 28 | 3 | 24 | 0.5 | ER+/N+ |
| 44 | 33 | 19 | 3 | 6 | 0.4 | ER+/N+ |
| 45 | 73 | 27 | 3 | 3 | 0.3 | ER+/N+ |
| 46 | 42 | 21 | 3 | 81 | 0.5 | ER+/N- |
| 47 | 72 | 18 | 2 | 10 | 0.4 | ER+/N- |
| 48 | 49 | 40 | 1 | 5 | 0.3 | ER+/N- |
| 49 | 77 | 23 | 3 | 39 | 0.5 | ER+/N+ |
| 50 | 78 | 28 | 1 | 22 | 0.8 | ER+/N- |
| 51 | 83 | 28 | 2 | 1 | 0.1 | ER+/N- |
| 52 | 78 | 21 | 2 | 2 | 1.2 | ER+/N- |
| 53 | 61 | 19 | 2 | 42 | 0.5 | ER+/N+ |
| 54 | 51 | 30 | 3 | 14 | 0.4 | ER-/N+ |
| 55 | 71 | 15 | 3 | 816 | 17.9 | ER+/N+ |
| 56 | 66 | 13 | 3 | 14 | 0.4 | ER+/N+ |
| 57 | 67 | 15 | 1 | 26 | 0.5 | ER+/N- |
| 58 | 79 | 21 | 3 | 17 | 0.1 | ER-/N- |
| 59 | 67 | 30 | 2 | 10 | 0.4 | ER+/N- |
| 60 | 75 | 30 | 2 | 10 | 2.0 | ER+/N- |
| 61 | 78 | 12 | 2 | 22 | 0.6 | ER+/N- |
| 62 | 75 | 99 |  | 5 | 0.2 | ER-/N- |
| 63 | 44 | 150 | 2 | 15 | 2.0 | ER-/N+ |
| 64 | 77 | 14 | 2 | 7 | 0.2 | ER-/N+ |
| 65 | 76 | 13 | 3 | 3 | 0.6 | ER+/N- |
| 66 | 45 | 20 | 2 | 94 | 21.4 | ER+/N- |
| 67 | 39 | 40 | 3 | 50 | 0.4 | ER+/N+ |
| 68 | 68 | 18 | 3 | 5 | 0.8 | ER-/N- |
| 69 | 50 | 18 | 3 | 7 | 1.3 | ER-/N+ |
| 70 | 53 | 17 | 2 | 4 | 1.7 | ER+/N+ |
| 71 | 81 | 18 | 1 | 6 | 0.2 | ER+/N- |
| 72 | 62 | 21 | 3 | 1 | 0.3 | ER-/N- |
| 73 | 66 | 12 | 3 | 4 | 2.2 | ER+/N- |
| 74 | 76 | 60 | 3 | 10 | 0.7 | ER+/N+ |
| 75 | 41 | 15 | 3 | 33 | 2.2 | ER+/N- |
| 76 | 74 | 22 | 2 | 76 | 4.5 | ER+/N- |
| 77 | 83 | 50 | 2 | 30 | 0.6 | ER+/N- |
| 78 | 40 | 17 | 3 | 191 | 1.8 | ER+/N+ |
| 79 | 65 | 15 | 2 | 36 | 0.4 | ER+/N+ |
| 80 | 56 | 35 | 3 | 278 | 42.1 | ER-/N+ |
| 81 | 74 | 15 | 1 | 41 | 2.9 | ER+/N- |
| 82 | 47 | 20 | 2 | 338 | 10.6 | ER-/N- |
| 83 | 81 | 14 | 3 | 422 | 59.5 | ER-/N- |
| 84 | 79 | 50 | 3 | 3882 | 94.4 | ER+/N+ |
| 85 | 41 | 25 | 3 | 389 | 4.3 | ER+/N+ |
| 86 | 33 | 25 | 3 | 26 | 0.9 | ER-/N- |
| 87 | 62 | 15 | 1 | 50 | 10.1 | ER+/N- |
| 88 | 80 | 31 | 2 | 1065 | 13.4 | ER+/N+ |
| 89 | 75 | 19 | 3 | 18 | 0.6 | ER+/N- |
| 90 | 75 | 16 | 3 | 351 | 190.5 | ER+/N+ |
| 91 | 44 | 16 | 2 | 1201 | 212.1 | ER-/N- |
| 92 | 62 | 19 | 2 | 385 | 7.9 | ER+/N+ |
| 93 | 62 | 13 | 1 | 100 | 6.1 | ER+/N+ |
| 94 | 43 | 50 | 2 | 402 | 3.9 | ER+/N+ |
| 95 | 72 | 17 | 3 | 596 | 15.7 | ER+/N+ |
| 96 | 70 | 22 | 3 | 101 | 11.9 | ER-/N+ |
| 97 | 78 | 30 | 3 | 130 | 4.6 | ER-/N- |
| 98 | 63 | 23 | 2 | 249 | 4.3 | ER+/N- |
| 99 | 61 | 29 | 3 | 61 | 66.2 | ER-/N+ |
| 100 | 48 | 50 | 2 | 34 | 2.8 | ER+/N+ |
| 101 | 76 | 33 | 3 | 104 | 2.5 | ER+/N+ |
| 102 | 62 | 60 | 3 | 417 | 86.9 | ER-/N- |
| 103 | 62 | 20 | 1 | 122 | 12.0 | ER+/N+ |
| 104 | 65 | 30 | 3 | 4 | 0.8 | ER-/N+ |
| 105 | 47 | 55 | 2 | 36 | 1.8 | ER+/N+ |
| 106 | 78 | 38 | 3 | 160 | 4.3 | ER-/N- |
| 107 | 59 | 35 | 3 | 362 | 8.2 | ER+/N+ |
| 108 | 61 | 30 | 2 | 296 | 5.1 | ER+/N+ |
| 109 | 60 | 9 | 2 | 42 | 13.9 | ER+/N- |
|  |  |  |  |  |  |  |
| Control samples | | | | | | |
| 1 |  |  |  | 0.6 | 4.3 |  |
| 2 |  |  |  | 1.7 | 1.1 |  |
| 3 |  |  |  | 2.3 | 0.8 |  |
| 4 |  |  |  | 1.7 | 1.2 |  |
|  |  |  |  |  |  |  |
| Cell lines used in this study | | | | | | |
| MJ1105 control | | | | 31.0 | 1.3 |  |
| MJ1105 hCAP18 | | | | 82100 | 1.0 |  |
| ZR-75-1 | | | | 675.3 | n.d. |  |
|  | | | |  |  |  |
