# Supplementary material for: Human antimicrobial protein hCAP18/LL-37 promotes a metastatic phenotype in breast cancer
Source: Breast Cancer Res. 2009 Jan 30;11(1):R6. doi: 10.1186/bcr2221 (PMC2687709; doi:10.1186/bcr2221)
Supplement: Additional file 2 — A MS Word file containing a table that lists the transcription levels of hCAP18 and ERBB2 in mouse tumours. The values are relative to the MJ1105 control cell line as determined by RT-PCR, and normalised against total RNA as determined by 18S. [file bcr2221-S2.doc]

**Additional data file 2 .** Transcription levels of *hCAP18* and *ERBB2* in mouse tumors relative to the MJ1105 control line as determined by qPCR, and normalized against total RNA as determined by 18S.

| Tumors derived from control line | Expression *hCAP18* | Expression *ERBB2* |
| --- | --- | --- |
| Mouse 1 primary tumor | 1.7 | 0.7 |
| Mouse 2 primary tumor | 1.8 | 1.3 |
| Mouse 3 primary tumor | 2.2 | 0.9 |
| Mouse 4 primary tumor | 1.8 | 0.8 |
| Mouse 4 lymph node | 1.9 | 1.0 |
|  |  |  |
| Tumors derived from *hCAP18* transgenic line |  |  |
| Mouse 1 primary tumor | 931 | 0.8 |
| Mouse 2 primary tumor | 352 | 0.7 |
| Mouse 3 primary tumor | 535 | 0.8 |
| Mouse 3 lymph node 1 | 459 | 1.5 |
| Mouse 3 lymph node 2 | failed | failed |
| Mouse 4 primary tumor | 472 | 0.7 |
| Mouse 4 metastasis 1 | 755 | 0.8 |
| Mouse 4 metastasis 2 | 458 | 0.4 |
| Mouse 4 metastasis 3 | 965 | 0.8 |
| Mouse 4 lymph node | 657 | 0.6 |
| Mouse 4 ascites | 1783 | 1.4 |
| Mouse 5 primary tumor | 1526 | 1.0 |
| Mouse 5 lymph node | failed | failed |
| For comparison: MJ 1105 *hCAP18, in vitro* | 2650 | 0.9 |
