# Supplementary figures and images for: Human antimicrobial protein hCAP18/LL-37 promotes a metastatic phenotype in breast cancer
Source: Breast Cancer Res. 2009 Jan 30;11(1):R6. doi: 10.1186/bcr2221 (PMC2687709; doi:10.1186/bcr2221)

(a)

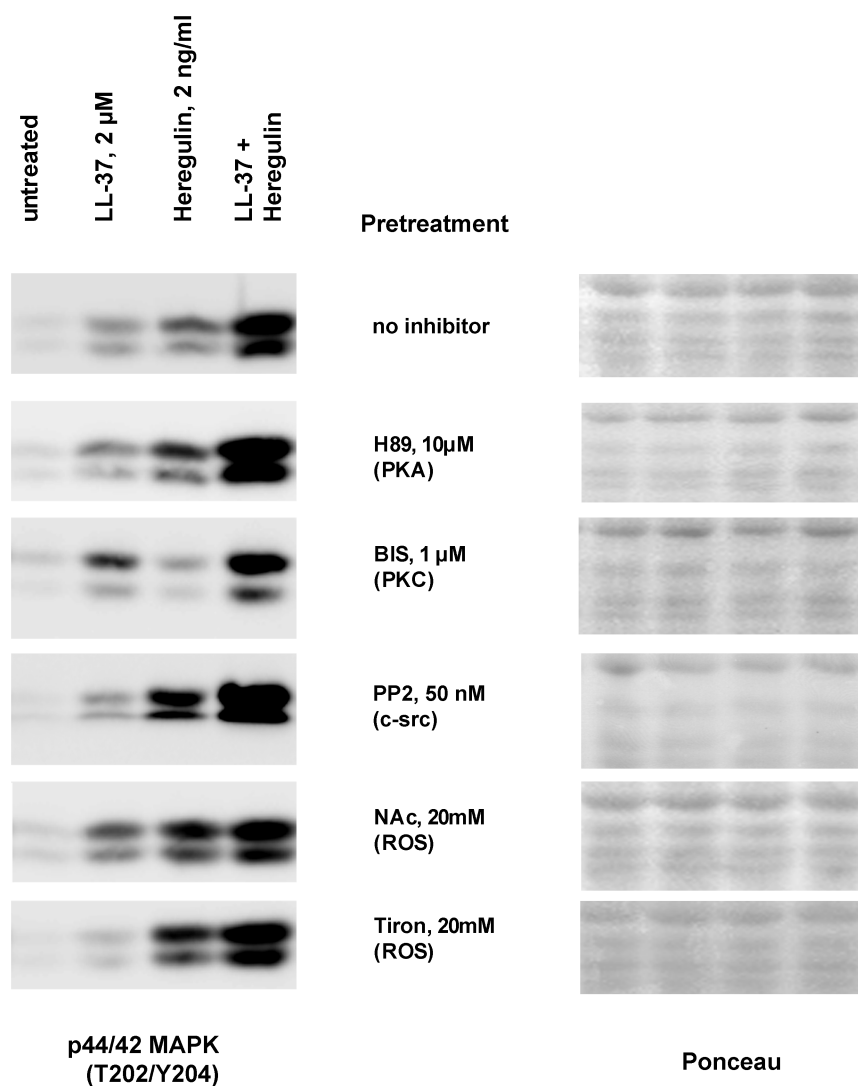

(b)

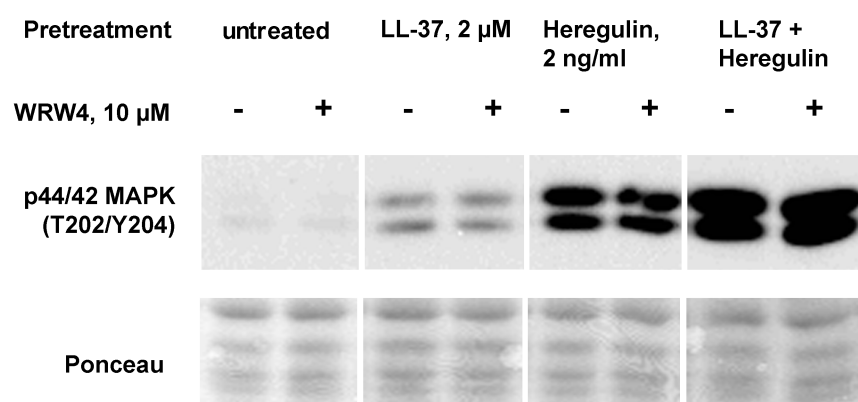

Supplement: Additional file 3 — A pdf file containing a figure of a Western blot analysis excluding cross-talk mechanisms for the synergistic effect of LL-37 and HRG on MAPK phosphorylation. (a) The analysis on ZR75-1 cells stimulated with LL-37 and/or HRG after 30 minutes pretreatment with inhibitors as indicated. The concentration of inhibitors is listed together with their prime targets. (b) The evaluation of the effect of WRW4, untreated samples being run side-by-side with treated samples to exclude even minimal differences. All experiments were run in triplicates (n = 3), and repeated at one independent occasion. [file bcr2221-S3.pdf]

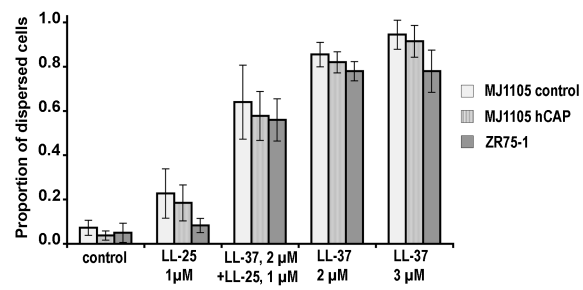

Additional data file 4

Supplement: Additional file 4 — A pdf file containing a figure displaying the rate of morphological changes in soft agar clones from ZR75-1, MJ1105 control and hCAP18 transgenic cells, as evaluated in Figure 3 (n = 4). [file bcr2221-S4.pdf]

**Primary tumors**

**metastases/lymph nodes**

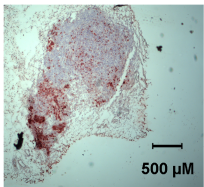

**MJ1105 hCAP derived tumors**

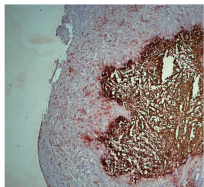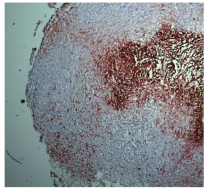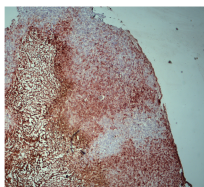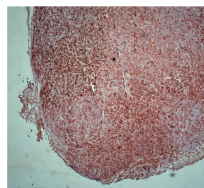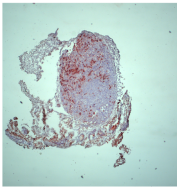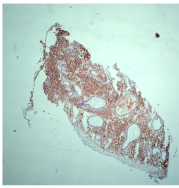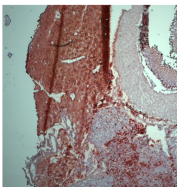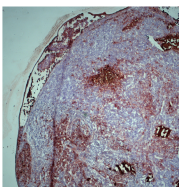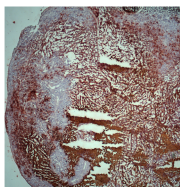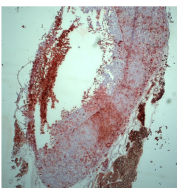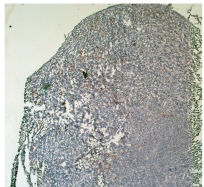

**control tumors**

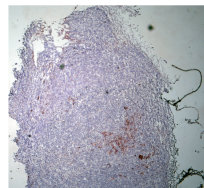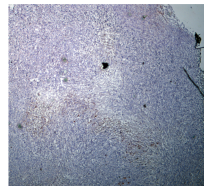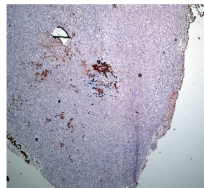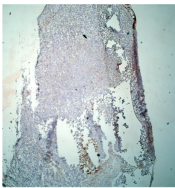

Supplement: Additional file 5 — A pdf file containing a figure showing the immunohistochemical analysis with anti-LL-37 antibodies on all mouse tumours in this study. [file bcr2221-S5.pdf]
